# Current status of primary hyperoxaluria type 1 in Japan

**DOI:** 10.1007/s00240-025-01931-w

**Published:** 2026-01-16

**Authors:** Tomohide Ogawa, Keita Okamoto, Mao Yamamoto, Yuya Sato, Kei Ushijima, Daisuke Numahata, Hideki Takeshita, Hiromu Inai, Mizuki Onozawa, Jun Miyazaki, Tatsuya Takayama

**Affiliations:** 1 Department of Clinical Medical Sciences, International University of Health and Welfare, Graduate School of Medicine, Chiba, Japan; 2 Japan Hereditary Urolithiasis Research Group (J-HUG), Fukuoka, Japan; 3 Department of Urology, International University of Health and Welfare Hospital, Tochigi, Japan; 4 Department of Urology, International University of Health and Welfare Narita Hospital, Chiba, Japan

**Keywords:** Primary hyperoxaluria, Alanine:glyoxylate aminotransferase, RNA interference, Oxalosis

## Abstract

Primary hyperoxaluria (PH) is a rare autosomal recessive disorder characterized by an abnormal increase in urinary oxalate excretion. Primary hyperoxaluria type 1 (PH1), the most common PH subtype, is caused by a deficiency of the liver-specific enzyme alanine:glyoxylate aminotransferase, which is localized to peroxisomes. In Japan, the status of PH1 has not been studied in two decades, and few longitudinal studies of PH1 patients have been conducted worldwide. This study aims to analyze the present situation regarding PH1 in Japan. We conducted a literature review of PH1 cases reported in Japan between 2003 and 2023. Twenty patients were diagnosed with PH1 during the study period, including 7 males, 12 females, and 1 patient with an unspecified sex. The median ages at symptom onset and diagnosis were 4.21 years (range: 0.17–47) and 5.5 years (range: 0.17–43), respectively. Ten patients (50%) were diagnosed before age 18, four (44%) of whom had already progressed to end-stage renal disease (ESRD). Among the six patients diagnosed after 18 years, all had ESRD or progressed to ESRD soon after diagnosis. The overall survival (OS) rate was 81% at 5, 10 and 20 years post-diagnosis. The mean observation period was 5.62 years (range: 0–32.6). Few longitudinal studies on PH1 patients have been conducted in Japan or worldwide. Over the past 20 years, the median ages at both symptom onset and diagnosis have significantly decreased, and the OS rate has increased. Early diagnosis and intervention may contribute to better outcomes for PH1 patients in Japan.

**Supplementary Information:**

The online version contains supplementary material available at 10.1007/s00240-025-01931-w.

## Introduction

Primary hyperoxaluria (PH) is a rare autosomal recessive disorder characterized by excessive increases in urinary oxalate levels. Primary hyperoxaluria type 1 (PH1), the most common subtype of PH, results from a deficiency of the liver-specific enzyme alanine:glyoxylate aminotransferase (AGT), which is localized in peroxisomes [1]. AGT is also known as serine:pyruvate aminotransferase (SPT). SPT/AGT is a bifunctional enzyme that detoxifies glyoxylate by converting it to glycine and plays a significant role in serine metabolism [2]. A functional defect in AGT leads to elevated blood concentrations of both oxalate and glycolate, resulting in increased urinary excretion of these metabolites. As renal function declines and the glomerular filtration rate falls below 30–40 ml/min/1.73 m^2^, urinary oxalate excretion decreases but also plasma oxalate increases, leading to systemic oxalosis, such as calcium oxalate deposition in the heart, blood vessels, joints, bones, retina and other organs [3]. PH1 accounts for 80% of all PH cases and is considered the most severe subtype. Many patients progress to end-stage renal disease (ESRD) in early childhood, and approximately half of patients develop ESRD by the age of 40 [4]. Until recently, liver and kidney transplantations were the only curative treatments for PH1. However, novel therapeutic agents targeting metabolic enzymes, such as RNA interference (RNAi), have been developed [5, 6]. Clinical trials are currently underway in Japan.

Based on our previous study, in Japan, 59 PH1 cases were reported between 1962 and 2003 [7]. However, the current status of PH1 has not been systematically studied over the past two decades, and a comprehensive overview of Japanese PH1 patients is lacking. The aim of this study was to conduct a longitudinal analysis of PH1 cases in Japan, which has rarely been performed globally, and to improve the clinical and epidemiological understanding of PH1 among the Japanese population in the era preceding the widespread implementation of RNAi treatments.

## Methods

We conducted a retrospective review of published medical literature on patients diagnosed with PH1 in Japan between 2003 and 2023. A total of 211 journals were screened using Ichushi-Web, a comprehensive database of Japanese medical literature. We also searched by using PubMed, but did not find any papers describing the clinical course of individual PH1 cases, except for cases that overlapping with those found on Ichushi-Web.

Articles pertaining to primary hyperoxaluria type 2 (PH2) were excluded, along with duplicate PH1 case reports. No reports on primary hyperoxaluria type 3 (PH3) have been confirmed in Japan. Ultimately, 20 PH1 cases were identified and included in this study.

## Results

**Fig. 1:**
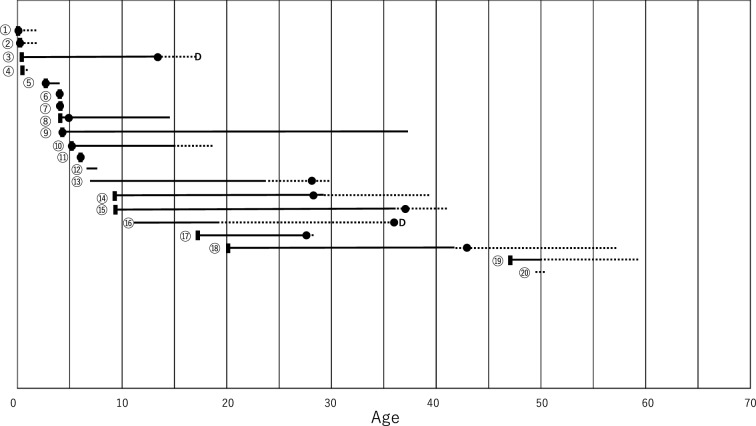
Age at symptom onset and subsequent clinical course of 20 Japanese patients with PH1. ❚, age at symptom onset; ●, age at diagnosis; D, death. ━(Solid line), period of observation without renal replacement therapy. (Dotted line), period of renal replacement therapy (including dialysis and kidney transplantation). The time interval between ❚ and ● represents the diagnostic delay. The circled numbers represent patient identification numbers. Data on age at symptom onset were missing in four cases (④, ⑫, ⑲ and ⑳). Data on age at diagnosis were missing in four cases (⑫, ⑬, ⑯ and ⑳). Three patients developed infantile oxalosis (①, ② and ④). Thirteen patients developed ESRD except 7 patients (⑤, ⑥, ⑦, ⑧, ⑨, ⑪ and ⑫). Pyridoxine (vitamin B6) was administered to seven patients (①, ③, ⑤, ⑧, ⑨, ⑮ and ⑲). ⑫, the short horizontal bar indicates the time of liver transplantation, as there were no other details about age. ⑬, The exact age at symptom onset was unknown, but it was reported as “childhood”; thus, for convenience, we used the mean childhood age of 7 years (range: 3–11 years) for Fig. 1 only. ⑯, The exact age of symptom onset was unknown. However, it was reported as “pediatric”; therefore, for convenience, we used the mean pediatric age of 11 years (range: 7–15) for this Fig. 1 only. ⑲, the age at symptom onset was 47 years old, but the age of diagnosis is unknown. This is one reason why the range of ages for symptom onset is broader than the range of ages for diagnosis. ⑳, the dotted line indicates the age at the time of examination, as there are no other details regarding the clinical course

A total of 20 patients were diagnosed with PH1 during the study period. We identified 20 patients, including 7 males, 12 females, and 1 patient with an unspecified sex. The median age at symptom onset was 4.21 years (range: 0.17–47), and the number of patients that account for the range is 16. The median age at diagnosis was 5.5 years (range: 0.17–43), and the number of patients accounting for the range is 16. The age at symptom onset and subsequent clinical course are shown in Fig. 1. Nine patients (45%) presented with initial symptoms before the age of 5, and six of these (66.7%) were diagnosed before the age of 5. Three patients developed infantile oxalosis, and it accounted for 15% (3 cases out of 20). Two patients (10%) presented with symptoms after age 18, including one whose first symptoms appeared at age 47. Ten patients (50%) were diagnosed before age 18, four (40%) of whom had already progressed to ESRD at diagnosis. In contrast, six patients (30%) were diagnosed after age 18, and all six (100%) had ESRD at the time of diagnosis or progressed to ESRD soon after diagnosis. Among the 20 patients, thirteen (65%) developed ESRD and needed renal replacement therapy. Eleven patients developed nephrocalcinosis only or nephrocalcinosis with urinary stones, and nine of the 11 patients (81.8%) progressed to ESRD (Supplementary Table 1).

Diagnostic methods included urinary measurements of oxalate and glycolate, serum analysis, genotyping, liver biopsy, bone biopsy, autopsy, stone composition analysis, and clinical evaluation. The diagnostic methods used for each patient are summarized in Supplementary Table 2A, and only available measurements, such as urinary oxalate, urinary glycolate, serum oxalate and serum glycolate, are summarized in Supplementary Table 2B for reference. Five patients were diagnosed by genotyping, although details were unavailable for three. Among the two documented patients, one patient was homozygous for a nonsense mutation (p.Arg111X), and the other patient was compound heterozygous for c.751_752delTGinsAA and C.1093.G > T. The c.751_752delTGinsAA mutation is novel, and functional analysis confirmed a complete loss of AGT activity [8, 9]. Liver biopsy was performed in seven patients to measure AGT activity, and a decrease in AGT activity was confirmed in the seven patients. In two cases, further analysis, immunohistochemical analysis, was performed for mistargeting of AGT. Immunohistochemical analysis confirmed the mistargeting of AGT to the mitochondria and cytoplasm instead of the peroxisome in the two patients. Details of whether immunohistochemical analysis was performed on the remaining five patients who underwent liver biopsy are unknown. Bone biopsy and autopsy were performed in two and one patients, respectively. Autopsy revealed calcium oxalate crystal depositions in various organs throughout the body. Four patients were diagnosed based on elevated urinary oxalate and glycolate levels. Three of these patients were sisters, and two were twins. Stone composition analysis confirmed that calcium oxalate was the primary component. Diagnostic details were unavailable for the remaining two patients due to insufficient information.

**Fig. 2:**
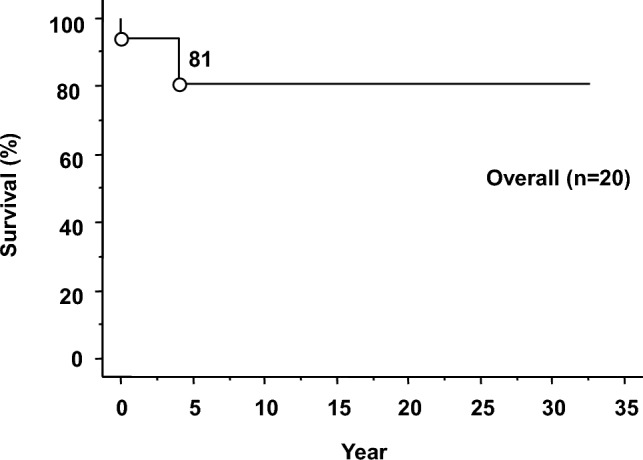
Overall survival rate of Japanese patients with PH1 postdiagnosis. Kaplan‒Meier curve showing the overall survival rate (%) after diagnosis in 20 patients with PH1. The survival rate was 81% at 5, 10, and 20 years post-diagnosis

In terms of transplantation medicine, preemptive liver transplantation was performed in two patients. Two additional patients underwent liver transplantation and are waiting for kidney transplantations. One patient died from sepsis 47 days after deceased-donor liver transplantation. Combined liver–kidney transplantation was performed in five patients between 2003 and 2023, all of whom had favorable outcomes (Supplementary Table 3). One patient underwent isolated kidney transplantation abroad at age 49 following recurrent stone formation from age 20 and the initiation of peritoneal dialysis and hemodialysis at age 42. More than 10 years post-transplantation, the patient remains alive. The overall survival (OS) of the 20 patients following diagnosis is shown in Fig. 2. The OS rates were 81% at 5, 10, and 20 years. The mean observation period was 5.62 (range: 0–32.6).

## Discussion

The estimated prevalence of PH is approximately 1–3 per 1,000,000 individuals [10]. According to our previous research, in Japan, 59 patients were diagnosed with PH1 between 1962 and 2003 [7]. In the present study, we confirmed 20 additional cases of PH1 between 2003 and 2023. Furthermore, a review of published literature from 1962–2003 revealed six articles describing seven PH1 patients who were not included in the earlier dataset. Thus, a total of 86 PH1 cases have been identified in Japan to date. Considering the estimated prevalence, more potential patients with PH1 exist in Japan due to underdiagnosis.

The age at onset of PH1 in Japan was previously higher than that reported in Western countries [7]. However, our current findings indicate a significant shift over the past two decades. The median age at symptom onset has decreased from 13.0 years (range: 0–58) to 4.21 years (range: 0.17–47), which is comparable to those in Western cohorts. Similarly, the median age at diagnosis decreased from 17.0 years (range: 0.02–63) to 5.5 years (range: 0.17–43). The previous cohort included 59 patients, but the current cohort had a small sample size of fewer than 20. For reference, we calculated the median age at symptom onset and diagnosis for both cohorts. The median age at symptom onset combining data from both cohorts was 6 years (range: 0–58), and the sample size was 75. The median age at diagnosis was 16 years (range: 0.02–63), and the sample size was 75.

The time lag between symptom onset and diagnosis is known as the diagnostic delay. This delay is a recognized challenge in PH1 and affects both developing and developed countries. Globally, the average diagnostic delay is estimated to be approximately 5 years [11]. In our study, the average diagnostic delay in Japan was 6.75 years (range: 0–28), slightly exceeding the global average. Diagnostic delay tends to increase with older age at symptom onset. Despite improvements in early recognition, delays persist, likely owing to the rarity of PH1, limited clinical awareness, the heterogeneity of symptoms and variability in age at onset.

The clinical course of PH1 varies across regions. We reviewed long-term studies (≧14 years) from other countries and summarized the data in Table 1. Compared with patients in other countries, Japanese patients exhibit a broader age range at onset and diagnosis. Despite Japan’s larger population, its annual incidence rate remains lower—approximately one case per year—than the three cases annually reported in other nations. Notably, the prevalence of PH1 is higher in regions such as Kuwait and Tunisia, which have higher rates of consanguineous marriages [12, 13]. In this study, parental first-cousin marriage was documented in two of the 20 cases.

**Table 1:** Epidemiological characteristics of PH1 across countries

Refs.	Country	Median age at onset (Age range)	Median age at diagnosis (Age range)	Sex ratio		Number of PH1 cases	Publication year	Years of study (Study period)	Annual incidence rate
				Male	Female				
[14]	USA	9	12	N/A	N/A	75	2003	28 years (1970–1998)	2.68
[15]	The Netherlands	6.0 (0–50)	7.3 (0–57)	27 (47.4)	30 (52.6)	57	2003	18 years (1985–2003)	3.17
[7]	Japan (previous study)	13 (0–58)	17 (0.02–63)	35 (59)	24 (40.7)	59	2005	41 years (1962–2003)	1.44
[16]	Tunisia	N/A	5.75 (0.25–14)	24 (54.5)	20 (45.5)	44	2012	14 years (1995–2009)	3.14
[17]	European 8 countries	3.9 (0–66)	8.1 (0–72)	300 (57)	226 (43)	526	2014	30 years (1980–2010)	2.19
[18]	Libya	5 (0.17–10)	N/A	1.3 (57)	1 (43)	60	2020	20 years (1998–2018)	3.0
[19]	Italy	4.0 (0.5–12.0)	9.9 (3.1–29.0)	61 (64.2)	34 (35.8)	95	2022	28 years (1992–2020)	3.39
[20]	China	4.25	N/A	2.69 (73)	1 (27)	48	2023	18 years (2004–2022)	2.67
	Japan (this study)	4.21 (0.17–47)	5.5 (0.17–43)	7 (35)	12 (60)	20	2023	20 years (2003–2023)	1

Abbreviations: N/A, not available. Note: The database for European countries includes data from eight European countries (France, Germany, Italy, the Netherlands, Poland, Spain, Sweden, and the United Kingdom). Regarding the incidence rate in European countries, the rate was divided by eight since the data from eight countries were merged. Because the start date of the American survey before 1970 was unclear, 75 cases from 1970–1998 were compiled in Table 1

Renal manifestations, particularly nephrocalcinosis and urolithiasis, were prominent among patients with PH1. The prognostic data are detailed in Supplementary Table 1. In this study, all four patients with nephrocalcinosis only progressed to ESRD. Among the seven patients with both nephrocalcinosis and urolithiasis, five (71.4%) developed ESRD. In our previous study, all 16 patients with nephrocalcinosis only and 16 of 18 with both nephrocalcinosis and urolithiasis progressed to ESRD [7]. These findings reinforce the strong association between nephrocalcinosis and renal failure [10, 15, 21, 22]

Compared with their Western counterparts, Japanese patients with PH1 tend to have milder symptoms [7]. One hypothesis attributes this phenomenon to dietary habits [23]. Studies have shown that compared with African Americans, Caucasians have higher rates of nephrolithiasis and hyperoxaluria, even under controlled oxalate-rich diets [24, 25]. Genetic factors influencing intestinal oxalate absorption may contribute to these differences. Additionally, obesity is associated with increased urinary oxalate excretion in both sexes, and compared with nonobese individuals, obese stone formers tend to have higher oxalate levels [26].

Until recently, liver and kidney transplantation were the only curative treatments for PH1. However, the development of RNAi therapies targeting glyoxylate metabolism has ushered in a new era of treatment. Clinical trials are currently underway in Japan, and the approval of siRNA-based therapies could significantly improve outcomes. Nevertheless, challenges such as diagnostic delay and underdiagnosis persist. To address these issues, we established the Japanese Hereditary Urolithiasis Research Group (J-HUG), a nonprofit organization dedicated to building a national PH registry and identifying additional patients.

This study is limited by the retrospective nature of the literature review, which resulted in incomplete patient data for some cases. The missing information included age, sex, symptom onset, diagnostic method, transplantation timing and prognosis. Establishing a national registry may help resolve these issues.

In conclusion, there have been few longitudinal studies related to patients with PH1 not only in Japan but also globally. Compared with our previous study, which was conducted 20 years ago, the median ages at onset and diagnosis have significantly decreased, and OS rate has improved. However, diagnostic delays in Japan remain slightly longer than the global average. Reducing these delays is essential for improving the prognosis of patients with PH1.

## Supplementary Information

Below is the link to the electronic supplementary material.

Supplementary file1

## Data Availability

The data that support findings of this study are available from the first author [T.O.] and corresponding author [T.T.] upon reasonable request.
