# Supplementary material for: Current status of primary hyperoxaluria type 1 in Japan
Source: Urolithiasis. 2026 Jan 16;54(1):34. doi: 10.1007/s00240-025-01931-w (PMC12811274; doi:10.1007/s00240-025-01931-w)
Supplement: Supplementary file 1 — Supplementary file1 [file 240_2025_1931_MOESM1_ESM.docx]

**Supplementary Table 1.** Relationships between renal manifestations and ESRD in patients with PH1.

| Manifestations | Patients (%) | Patients with ESRD (%) |
| --- | --- | --- |
| NC | 4 (20) | 4/4 (100) |
| NC with UL | 7 (35) | 5/7 (71.4) |
| UL | 7 (35) | 3/7 (42.9) |
| No NC, no UL | 0 (0) | 0 (0) |
| Unknown | 2 (10) | 1/2 (50) |
| Total number | 20 | 13 (65) |

Abbreviations: NC, nephrocalcinosis; UL, urolithiasis.

Notes:

This table details the proportion of patients who developed ESRD relative to their initial renal manifestations.

**Supplementary Table 2A.** Diagnostic examinations performed in 20 patients with PH1

| Ref. | Patient No. | Urinary oxalate | Urinary glycolate | Serum oxalate | Serum glycolate | Genotyping | Liver biopsy | Bone biopsy | Autopsy |
| --- | --- | --- | --- | --- | --- | --- | --- | --- | --- |
| [1] | ① | ● | ● | ● |  |  | ● |  |  |
| [2] | ② |  |  | ● | ● | ● |  |  |  |
| [3] | ③ | ● | ● |  |  |  | ● |  |  |
| [4] | ④ | ● | ● | ● | ● | ● |  |  |  |
| [5] | ⑤ | ● | ● | ● |  |  | ● |  |  |
| [6] | ⑥ | ● | ● |  |  |  |  |  |  |
| [6] | ⑦ | ● | ● |  |  |  |  |  |  |
| [7] | ⑧ | ● |  | ● |  | ● | ● |  |  |
| [8] | ⑨ | ● | ● |  |  | ● |  |  |  |
| [9] | ⑩ | ● |  |  |  |  |  |  |  |
| [6] | ⑪ | ● | ● |  |  |  |  |  |  |
| [10] | ⑫ | N/A |  |  |  |  |  |  |  |
| [11] | ⑬ |  |  |  |  |  |  | ● |  |
| [12] | ⑭ | N/A |  |  |  |  |  |  |  |
| [13] | ⑮ |  |  |  |  |  | ● |  |  |
| [14] | ⑯ |  |  |  |  |  |  |  | ● |
| [12] | ⑰ |  |  |  |  |  | ● |  |  |
| [15] | ⑱ | ● | ● | ● | ● |  | ● |  |  |
| [16] | ⑲ |  |  |  |  |  |  | ● |  |
| [17] | ⑳ |  |  |  |  | ● |  |  |  |

Abbreviations: N/A, not available.

Notes:

This table summarizes the diagnostic modalities used across 20 patients with confirmed PH1.

The circled numbers represent patient identification numbers and match those in Figure 1.

**Supplementary Table 2B.** Oxalate and glycolate measurements in patients with PH1

| Ref. | Patient  No. | Urinary oxalate | Urinary glycolate | Serum  oxalate | Serum  glycolate |
| --- | --- | --- | --- | --- | --- |
| [1] | ① | 0.17 mmol/mmol creatinine  (standard value < 0.1 mmol/mmol) | 0.28 mmol/mmol creatinine  (standard value for 1-4 y/o  < 0.09 mmol/mmol) | 100 µmol/L  (standard value  1.31-30.8 µmol/L) | N/A |
| [2] | ② | N/A | N/A | 42.2 µmol/L  (standard value  < 1-5 µmol/L) | 61.8 µmol/L  (standard value  < 5 µmol/L) |
| [5] | ⑤ | 11.0 mg/kg/day  (standard value ≦ 0.57 mg/kg/day) | 5.1 mg/kg/day  (standard value ≦ 0.35 mg/kg/day) | 2.34 mg/L  (standard value  1.3±0.7 mg/L) | N/A |
| [8] | ⑨ | 120 mg/day  (standard value < 40 mg/day) | 86 mg/day  (standard value < 15-60 mg/day) | N/A | N/A |
| [9] | ⑩ | 270 mg/g creatinine  (standard value < 40-45 mg/g) | N/A | N/A | N/A |
| [15] | ⑱ | 1,665 µmol/day  (standard value < 350 µmol/day) | N/A | 130 µmol/L  (standard value  < 1-5 µmol/L) | 372 µmol/L  (standard value  < 5 µmol/L) |

Abbreviations: N/A, not available.

Notes:

For reference, only available measurements are summarized in this table.

**References (Supplementary Table 2A and 2B)**

1. Otsubo Y, Araki K, Mitsutake S, Okazaki N, Nakayama M, Tsutsumi Y, Nakashita S (2007) [A case of a boy with oxalosis undergoing maintenance dialysis since 2 months of age in preparation for combined liver and kidney transplantation] Kanjin fukugoishoku wo mezashi seigo 2kagetsu kara ijitosekichu no oxalosis no danji-rei (in Japanese). J Jpn Pediatr Soc (0001-6543) 111(7):888-892.

2. Mori Y, Tamamura S, Yamada K, Ooto T, Watanabe Y, Taniguchi Y, Hayashi T, Shigematsu Y (2015) Primary hyperoxaluria type 1 and end-stage renal disease in a 3-month-old infant. Jpn J Pediatr Nephrol (0915-2245) 28(1):60–67. https://doi.org/10.3165/jjpn.28.60

3. Miura K, Fujii H, Fujiki T, Matsumura H, Kuroda N, Tanaka E, Chikamoto H, Akioka Y, Kono M, Miyagawa S, Hattori M, Ogawa Y, Takayama T (2008) [A male infant with primary hyperoxaluria type 1 undergoing peritoneal dialysis with no obvious organ damage other than end-stage renal failure] Makki jinfuzen igai ni akiraka na zokishogai ga naku, fukumaku toseki wo okonatteiru genpatsusei koshusannyosho 1gata no danji-rei (in Japanese). Journal of Japanese Society for Pediatric Renal Failure (1341-5875) 28:105-106.

4. Tsuchida S, Kagatani R, Oyama Y, Kuwayama N, Tamura M, Narumi M, Tamura H, Noguchi A, Takahashi T. (2023) [Initial management of end-stage renal disease in infants with primary hyperoxaluria] Makki jinfuzen de hakken sareta genpatsusei koshusannyusho no nyuji eno shokitaio (in Japanese). Japanese Journal of Apheresis (1340-5888) 42 Suppl.:139.

5. Kinoshita D, Takanashi M, Komatsu H, Kudo N, Yutaka N, Wada N, Ikuta H. (2005) [A case of a girl with primary hyperoxaluria type 1 diagnosed early and treated with vitamin B6] Soki ni shindan ni itari, vitaminB6 ga yuko de atta genpatsusei koshusannyosho 1 gata no joji-rei (in Japanese). Japanese Journal of Pediatrics (0021-518X) 58(3):401-405.

6. Sakakibara O, Kamata A, Suzuki M, Sato K, Iijima M, Kinoshita K, Obinata K, Kaneko K, Morozumi M, Ogawa Y (2003) [A case of three sisiters with primary hyperoxaluria type 1] Genpatsusei koshusannyosho 1gata no 3 shimai-rei (in Japanese). J Jpn Pediatr Soc. (0001-6543) 107 (11):1574.

7. Terano C, Sakai T, Nagatani K, Shinozuka S, Okuda Y, Harada R, Nagaoka Y, Hamada R, Hataya H, Honda M, Kasahara M (2013) [A 10-year-old girl with primary hyperoxaluria underwent preemptive liver transplantation to preserve her native kidney] Koyujin kinoiji wo mokuteki ni senkoteki kanishoku wo okonatta genpatsusei koshusannyosho no 10sai joji (in Japanese). Journal of Japanese Society for Pediatric Renal Failure (1341-5875) 33:159-161.

8. Takayama T, Okuyama T, Nakamura K, Murayama K, et al. (2024) [Inborn Errors of Metabolism Clinical File (part 1 case series R.Peroxisomal disease 4.Primary Hyperoxaluria Type 1)] Senten taisha ijosho clinical file (in Japanese). Tokyo: SHINDAN TO CHIRYO SHA Inc.

9. Tamaki S, Sato H, Aoki Y, Itabashi Y, Matsui Y, Sakai T, Hamada R, Ishikura K, Hataya H (2014) [A case of primary hyperoxaluria: kidney transplantation immediately after liver transplantation saved the patient from renal failure due to inadequate understanding of the risk of renal failure] Jinfuzen risuku no rikai furyo niyori jinfuzen ni itari kanishokugosumiyakani jinishoku wo shiko shi kyumei shieta genpatsusei koshusannyosho no 1-rei (in Japanese) . The Japanese Urological Association 102:659.

10. Sasaki K, Sakamoto S, Uchida H, Hamano I, Shigeta T, Kanazawa H, Fukuda A, Takayama T, Nagata M, Kasahara M, Matsui A (2013) Living donor liver transplantation for primary hyperoxaluria type 1. Jpn J Pediatr Gastroenterol Hepatol Nutr (1346-9037) 27 Suppl.:150.

11. Yamashita Y, Yamamoto M, Kishi T, Otsuka S, Takashima T, Haraguchi S, Miyazono M, Ikeda Y, Sauchi T (2009) [A case of primary hyperoxaluria patient undergoing maintenance dialysis] Genpatsusei koshusannyosho ga utagwareta ijitoseki no 1-rei (in Japanese). Journal of Japanese Society for Dialysis Therapy (1340-3451) 42 Suppl.1:742.

12. Kato T, Kotera Y, Ohmori A, Yamashita S, Nemoto S, Ariizumi S, Katagiri S, Egawa H, Yamamoto M (2018) Single center experience of liver transplantation for Primary Hyperoxaluria Type 1. Japanese Society of Hepato-Biliary-Pancreatic Surgery 30:460.

13. Hori T, Kaido T, Tamaki N, Toshimitsu Y, Ogawa K, Uemoto S (2013) An adult with primary hyperoxaluria type 1 regrets not receiving preemptive liver transplantation during childhood: report of a case. Surg Today 43:1185-1187.

14. Hisanaga S, Nishiura R, Tokura K, Samejima N, Fujimoto S, Kuribayashi T (2008) [An autopsy case of primary hyperoxaluria complicated with secondary amyloidosis and death from end-stage renal failure after 18 years of dialysis] Genpatsusei koshusannyosho niyori makki jinfuzen tonari toseki 18nenme ni nijisei amylodosis wo gappei shi shibo shita bokenrei no hokoku (in Japanese). Journal of Japanese Society for Dialysis Therapy (1340-3451) 41 Suppl.1:758.

15. Shiraishi Y, Ishida H, Miyauchi Y, Shirakawa H, Shimizu T, Omoto K, Tanabe K (2010) Primary hyperoxaluria type 1 with renal transplantation alone: a case report of long-term follow-up with birateral bephrectomy and preventive drugs for urinary stone. Japanese Journal of Transplantation (0578-7947) 45(4):381-385.

16. Ohtani H, Gotoh H, Tada M (2016) Oxalosis showed hyperparathroidism-like findings. Akita Jinfuzen Kenkyukaishi (in Japanese) 19:125-134.

17. Akita K, Kumakura Y, Nakajima E, Iijima T (2019) [A case of intractable pain due to impaired lower limb blood flow in a patient with primary hyperoxaluria and congenital antithrombin III deficiency] Genpatsusei koshusannyosho・sentensei antithrombin III kessonsho gappei kanja no kashi ketsuryushogai niyoru nanchisei totsu no 1-shorei (in Japanese). Palliative Care Research (1880-5302) 14 Supp1:S325.

**Supplementary Table 3.** Eleven cases of transplantation

| Patient No. (age, sex) | Diagnostic examinations | Age at initiation of PD and/or HD | Age at transplant (Type of transplant, age at transplant) | Renal outcome |
| --- | --- | --- | --- | --- |
| ① (2 y/o, M)  ② (2 y/o, F)  ③ (17 y/o, M)  ④ (10 mos., F)  ⑧ (14 y/o, F) | Liver biopsy  Genotyping  Liver biopsy  Genotyping  Genotyping | PD at 2 mos.  PD at 3 mos.  HD at 13 y/o  PD at 7 mos.  Dialysis-free | LDLT 2 y/o LDKT 2 y/o 7.5 mos.  LDLT 7 mos.  LDLT 17 y/o  DDLT 17 y/o  LDLT 10 mos.  LDLT 10 y/o | His liver and kidney function were stable for 65 days post-transplant; alive  PD continued 1 year after LDLT and waiting for LDKT; alive  LDLT failed due to portal vein thrombosis. Died due to sepsis 47 days after DDLT  N/A  Her kidney function was stable for 4 years and 7 months after LDLT; alive |
| ⑩ (17 y/o, F)  ⑫ (7 y/o, F) | N/A  N/A | HD at 15 y/o  Dialysis-free | LDLT 15 y/o LDKT 15 y/o 5.5 mos.  LDLT 7 y/o | No oxalate deposition in transplanted kidney 2 years post-transplant; alive  Her kidney function was stable for 1 month after LDLT; alive |
| ⑭ (39 y/o, M) | N/A | PD at 29 y/o HD at 37 y/o | DDLKT 39 y/o | HD continued for 3 months post-transplant to reduce serum oxalate; alive |
| ⑮ (41 y/o, M) | Liver biopsy | HD at 36 y/o | LDLT 38 y/o LDKT 38 y/o 8 mos. | No dialysis required 3 years post-transplant; alive |
| ⑰ (28 y/o, F)  ⑱ (59 y/o, M) | Liver biopsy  Liver biopsy | HD at 27 y/o  PD at 42 y/o  HD at 44 y/o | LDLT 28 y/o LDKT 28 y/o 9 mos.  LDKT 49 y/o | HD successfully withdrawn post-transplant; alive  His kidney function was stable for 9 years after LDKT; alive |

Abbreviations: DDLKT, simultaneous deceased-donor liver and kidney transplantation; DDLT, deceased-donor liver transplantation; HD, hemodialysis; LDKT, living-donor kidney transplantation; LDLT, living-donor liver transplantation; mos., months; N/A, not available; PD, peritoneal dialysis; y/o, years old.

Notes:

The circled numbers represent patient identification numbers, which match those in Figure 1, Supplementary Table 2A and Supplemental Table 2B.

Patient ages refer to the time of last clinical observation.
